# Proteomics and metabolomics combined study on endopathic changes of water‐soluble precursors in Tan lamb during postmortem aging

**DOI:** 10.1002/fsn3.2780

**Published:** 2022-02-15

**Authors:** Chen Ji, Liqin You, Ruiming Luo

**Affiliations:** ^1^ 56693 School of Agriculture Ningxia University Yinchuan China; ^2^ 12680 School of Biological Science and Engineering North Minzu University Yinchuan China

**Keywords:** glycolysis, lamb meat, metabolomics, postmortem aging, proteolysis, proteomics, water‐soluble compounds

## Abstract

Tan lamb is highly recommended breed in China. It is of great significance to understand the underlying mechanism of how water‐soluble flavor precursors metabolize in Tan lamb muscles during the postmortem aging period. In this study, we investigated the muscle pH, lactate dehydrogenase (LDH) activity, and the variations in water‐soluble flavor‐related metabolites. The proteome changes were profiled to provide insights into the biochemical changes affecting accumulation of water‐soluble flavor precursors in different aging stages (days 0, 4, and 8). The results indicated that pH value considerably decreased from day 0 to day 4, and increased from day 4 to day 8 (*p* < .05). The activity of LDH significantly increased from day 0 to day 4, and decreased from day 4 to day 8 (*p* < .05). Postmortem glycolysis was activated in 4 days, which directly affected the variations in metabolic enzymes and triggered the accumulation of flavor‐related carbohydrates. The free amino acids accumulated due to hydrolysis of structural proteins, with 3‐hydroxy‐L‐proline, aspartic acid, and methionine increasing from day 0 to day 4, and aspartic acid, serine, threonine, tyrosine, phenylalanine, and D‐phenylalanine from day 4 to day 8. The inosine and hypoxanthine accumulated due to the degradation of ATP. The results of the present study provide insightful information, revealing the differences in biochemical attributes in Tan lamb muscles caused by postmortem aging.

## Graphical abstract

The proteome changes were profiled to provide insights into the biochemical changes affecting accumulation of water‐soluble flavor precursors in different aging stages (days 0, 4, and 8). Postmortem glycolysis was activated in 4 days, which directly affected the variations in metabolic enzymes and triggered the accumulation of flavor‐related carbohydrates. The free amino acids accumulated due to hydrolysis of structural proteins, with 3‐hydroxy‐L‐proline, aspartic acid, and methionine increasing from day 0 to day 4, and aspartic acid, serine, threonine, tyrosine, phenylalanine, and D‐phenylalanine from day 4 to day 8. The inosine and hypoxanthine accumulated due to the degradation of ATP.

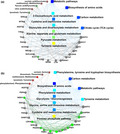

## 1 INTRODUCTION

Tan lamb, a unique breed in China, is popular among consumers for its tenderness, juiciness, and flavor. Postmortem aging improves meat quality effectively (Hopkins & Thompson, 2001; Kodani et al., 2017). The significant increase in the concentration of flavor precursors (carbohydrate‐related metabolites, adenine nucleotides degradation products, peptide, and free amino acids) was monitored in postmortem muscles (Koutsidis et al., 2008). It turns out that they react with other degradation products to form volatile compounds in charge of meat aroma (Castejón et al., 2015). The enhanced flavor of the dry‐aged beef loins could be due to the increase in amino acids, sugars, and nucleotides during aging (Kim et al., 2016).

Proteomics analysis provides a solid basis for the molecular modifications which muscles undergo during the course of conversion from muscle to meat (Paredi et al., 2012). Proteomics analysis was used to determine the postmortem tenderization rate of different horse muscles (della Malva et al., 2019), the differences in the proteomes of goat *longissimus thoracis* during freeze–thaw processing (Gu et al., 2020), and the biomarkers in Iberian wild deer (López‐Pedrouso et al., 2019). As a new subject, metabolomics reveals the complex biological regulative mechanism in the biological system through qualitative and quantitative analysis of the metabolites of a certain organism or cell in a specific period (Dixon et al., 2006). The integrated application of complementary proteomics and metabolomics analyzes the functions and regulative mechanisms of biomolecules in a comprehensive manner. As for the meat flavor, proteomics was performed to investigate the irradiation off‐flavor mechanism of goat meat, and 26 proteins involving seven functional terms were found to be related to off‐flavor (Jia et al., 2021). Metabolomics was used to evaluate grass‐fed beef loins aged in different methods, and greater number of protein‐ and nucleotide‐derived metabolites were observed in dry‐aged samples compared to wet‐aged and dry‐aged in bags samples (Setyabrata et al., 2021). MS‐based metabolomics was used to investigate the effects of castration on sheep muscles, the result of which highlighted that lipids and hydrophilic metabolites were affected by the castration, contributing partly to the improvement of the quality of mutton (Li et al., 2020).

When it comes to Tan lamb, no data are available on the integrated application of proteomics and metabolomics to characterize the effects of postmortem aging on the water‐soluble flavor precursors. In this study, the muscle pH and lactate dehydrogenase activity in postmortem Tan lamb muscles were investigated, and the proteome and metabolite profiles were examined using proteomic and metabolomics approaches. This study aimed to obtain more insights into the potential causes for the formation of water‐soluble flavor precursors during postmortem aging.

## 2 MATERIAL AND METHODS

### 2.1 Preparation of animals and samples

All the Tan lambs used for the study were grazed and raised under the same conditions. Eight Tan lambs (male, approximately 6 months old) were randomly selected and humanely slaughtered at a commercial meat company (Daxia Food Co. Ltd., Yanchi, Yinchuan, China) following Islamic practice and requirements of National Standards of PR China (GB 12,694–2016) without applying electric stimulation. The *longissimus dorsi* muscles were cut off immediately after slaughter, transferred to a polystyrene tray sealed with polyethylene films, and stored for 8 days (4ºC, relative humidity of 80%). A sterilized knife was used to collect samples (6 g) from the inside of *longissimus dorsi* at days 0, 4, and 8. The collected samples were immediately placed in a liquid nitrogen tank and transferred to a refrigerator at −80 ºC for further analysis. All the experiments were repeated with three independent biological replicates. The result of day 4 versus day 0 was recorded as Group 4/0, and day 8 versus day 4 as Group 8/4.

### 2.2 Detection of muscle pH

According to the method of Al‐Dalali et al. (2022), muscle pH was measured using a portable pH meter (Youke P611, Shanghai, China) after preparing a mixture of 5 g sample and 45 ml of distilled water (homogenized Jingxin LC‐11L, Shanghai, China) for 3 min. The pH meter was calibrated at pH 4.0, pH 6.86, and pH 9.18 (at 20°C) before the measurement of the samples.

### 2.3 Determination of lactate dehydrogenase (LDH) activity

LDH activities were surveyed with Elisa Kit (Fankew Industrial Co., Ltd, Shanghai, China) according to the manufacturer's instructions. Minced muscle (1 g) was lysed in 9 ml of phosphate‐buffered saline (PBS, pH 7.2, 0.01 mol/L) using a homogenizer (Jingxin JXFSTPRP‐24, Shanghai, China) at 4°C, and then centrifuged at 5,000 rpm at 4°C for 15 min, with the supernatant collected and the conjugate reagent (polygalacturonase, pectin methylesterase) serially diluted and added into a 96‐well microtiter plate. The supernatant was incubated at 37°C for 30 min with conjugate reagent (50 *µ*l), joined by chromogenic solution A (50 *µ*l, sodium acetate, citric acid, 30% H_2_O_2_) and B (50 *µ*l, EDTA‐Na, citric acid, glycerinum, tetramethylbenzidine), and incubated at 37°C in the dark for 15 min before adding the stop buffer (50 *µ*l, H_2_SO_4_, 1 mol/L). The enzymatic activity was obtained by testing the absorbance at 450 nm using a Microplate reader (LabSystem Multiskan, Finland). The serial dilution method was applied to dilute the conjugate reagent with PBS (0.01 mol/L) to draw the standard curve, and the LDH activities were calculated according to the standard curve.

### 2.4 Proteomic profiling of Tan lamb muscles by isobaric tags for relative and absolute quantitation (iTRAQ)

Each sample was accurately weighted for 5 g and transferred to a 1.5‐mL centrifuge tube with 500 *µ*l of sample lysate and a certain amount of protease inhibitor phenylmethanesulfonyl fluoride (PMSF) added to reach the final concentration of 1 mM. The muscle was homogenized and smashed by ice ultrasonic crushing for 3 min (twice, 40 kHz, 80 W, VCX130, Sonics, USA). The supernatant was collected after centrifugation (twice, 15,000 g, 15 min, Eppendorf, Germany) and mixed uniformly with 5‐fold precooled acetone. After being placed at −20°C overnight, the precipitation mixed well with chilled acetone was centrifuged (3 times, 4°C, 12,000 g, 15 min), dried at 25°C, and dissolved in the sample lysis solution (3 hr). The protein solution was obtained after centrifugation (twice, 25°C, 12,000 g, 10 min). Protein concentration was determined with the BCA method (Smith et al., 1985).

Protein digestion was performed according to the method of Wiśniewski et al. (2009). Following the digestion, a C18 columns and a vacuum concentration meter (Thermo, USA) were used to desalt and dry the peptides. The peptides labeling was conducted then by re‐dissolving the dried peptides powder for 100 *μ*l with 100 mM TEAB. The samples were labeled with 8 plex‐iTRAQ reagents (ABSCIEX, USA) as per the manufacturer's instructions. A Triple TOF 5600 Mass Spectrometer (SCIEX, USA) equipped with a Nanospray III source (SCIEX, USA) was utilized for proteomic profiling. Samples were loaded by a capillary C18 trap column (3 cm×100 µm, 3 µm, 150 Å, Eksigent) and then separated by a C18 column (15 cm×75 µm, 3 µm, 120 Å, Eksigent). The flow rate was 300 *µ*l/min and the linear gradient was 90 min (from 5%–85% B over 67 min; mobile phase A = 2% ACN/0.1% FA and B = 95% ACN/0.1% FA).

Analyst TS 1.1 software (Thermo Fisher Scientific, Waltham MA, USA) was used to analyze the raw data of proteomics. Protein pilot 5.0 software (*Ovis aries*. fasta) was applied to identify and quantify proteins. Those with FDR ≤1%, PSMs <40%, and protein score ≥1.3 were selected as highly reliable proteins, and proteins were considered as differentially abundant proteins with a fold change (FC) ≥ 2 or ≤0.5 and *p* < .05.

### 2.5 Identification of changes in water‐soluble flavor precursors of Tan lamb meat during postmortem aging

The sample derivation was completed following the experimental protocols of You and Luo (2019). Each sample (30 mg) was transferred to a 1.5‐mL Eppendorf tube, treated with 2‐chloro‐l‐phenylalanine (20 *μ*l, 0.3 mg/ml, Hengbai Biotechnology Co., Ltd., Shanghai, China) in methanol (CNW Technologies GmbH, Düsseldorf, Germany), and ground (60 HZ, 120 s) after being stored at −80 ºC for 2 min, and added with 120 *μ*l of chloroform. Following an ultrasonic‐associated extraction (25°C, 10 min), the samples were stored at 4 ºC for 10 min, and centrifuged at 12,000 rpm at 4 ºC for 10 min. Quality control samples were made by mixing aliquots of all samples to form pooled samples. An aliquot of 300 *μ*l of supernatant was transferred to a glass sampling vial, vacuum dried at room temperature, and joined by methoxyamine hydrochloride (80 *μ*l, 15 mg/ml, dissolved in pyridine, CNW Technologies GmbH, Düsseldorf, Germany). The resultant mixture was vortexed heavily for 120 s and cultivated at 37 ºC for 90 min. The BSTFA (80 *μ*l, with 1% TMCS, CNW Technologies GmbH, Düsseldorf, Germany) and n‐hexane (20 *μ*l, CNW Technologies GmbH, Düsseldorf, Germany) were added in and vortexed heavily for 120 s, and derivatized at 70 ºC for 1 hr. Then, the samples were incubated at 25 ºC for 30 min. A Pegasus 4D GC×GC‐TOF‐MS (LECO, St. Joseph, USA) was used for analyzing the derivatized samples.

NIST 11 and Fiehn database were used to identify and quantify the differential metabolites. The data were analyzed using SIMCA software package (14.0, Umetrics, Umea, Sweden). The Hotelling's T2 region, shown as an ellipse in score plots of the models, defined the 95% confidence interval of the modeled variation. Variable importance in the projection (VIP) ranked the overall contribution of each variable to the OPLS‐DA model and the variables with “VIP >1.00” and “*p* < .05” were considered relevant for group discrimination.

### 2.6 Bioinformatic analysis

Gene Ontology (GO) annotation of differentially abundant protein was performed using OmicsBean platform (http://www.omicsbean.cn/). The pathway analysis of differentially abundant protein and differential metabolite was queried against Kyoto Encyclopedia of Genes and Genomes (KEGG) pathway database (http://www.genome.jp/kegg/).

### 2.7 Integrative analysis

The KEGG pathways were selected as the carrier to map the differential proteins and differential metabolites to elucidate the effects of postmortem aging on meat flavor precursors, only the pathways with *p* < .05 were considered to have significant enrichment. Metabolite–protein interactions control a variety of cellular processes, to further elucidate the internal relationships between differentially abundant proteins and differential metabolites. The Pearson correlation coefficient was calculated according to the expression or relative content, with the interaction network built using R software (version 3.6.3).

### 2.8 Statistical analysis

The mean of the three samples was recorded and expressed in the form of the mean ±standard deviation (*SD*). The data were analyzed by IBM SPSS 26.0 (SPSS Inc., Chicago, IL, USA). A one‐way analysis of variance (ANOVA) was used to determine statistical significance, where *p* < .05 was considered as significant difference.

## 3 RESULTS

### 3.1 Muscle pH and LDH activity

**FIGURE 1:**
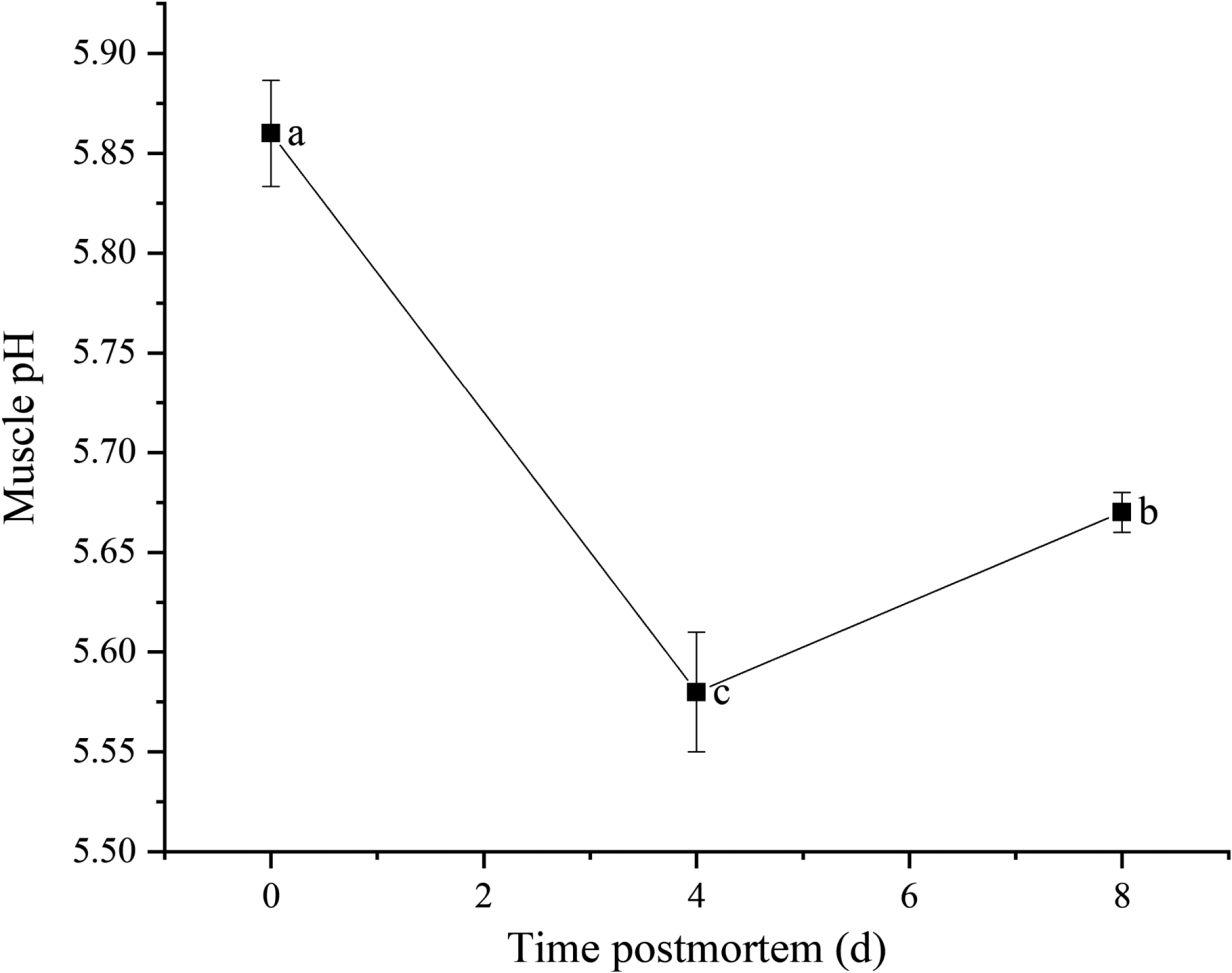
Changes in pH value of Tan lamb muscles. Note: Data are expressed in the form of mean ± *SD* (*n* = 3), a‐b referring to significant difference (*p* < .05)

The pH value of Tan lamb muscles conditioned at 4 ºC considerably decreased in the first 4 days (*p* < .05) and increased thereafter (*p* < .05). The minimum value was observed at day 4 and the maximum value immediately after slaughter (Figure 1).

**FIGURE 2:**
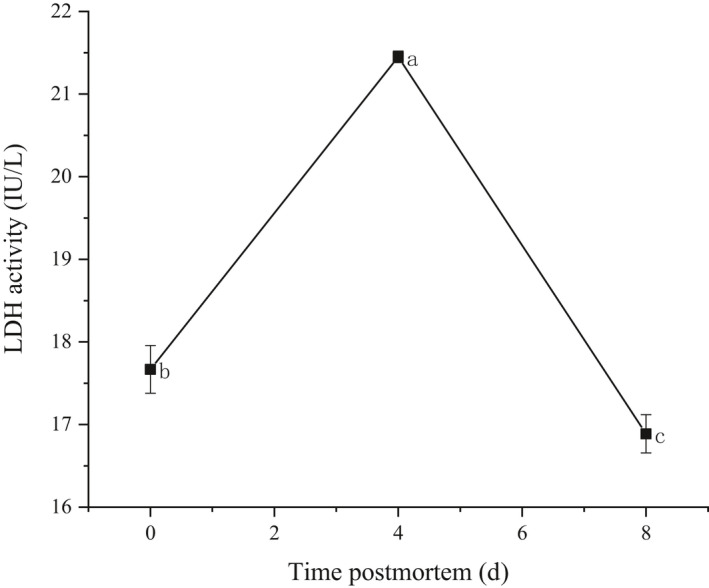
Changes in LDH activities on Tan lamb muscles. Note: Data are expressed in the form of mean ± *SD* (*n* = 3), a‐c referring to significant difference (*p* < .05)

As indicated in Figure 2, LDH activity of Tan lamb muscles significantly increased from day 0 to day 4 postmortem with the maximum value recorded at day 4 (21.45 IU/L), and decreased from 21.45 IU/L to 16.89 IU/L after day 4 (*p* < .05) with the minimum value recorded at day 8 (16.89 IU/L).

### 3.2 Protein profiles of Tan lamb muscles at different aging stages

**FIGURE 3:**
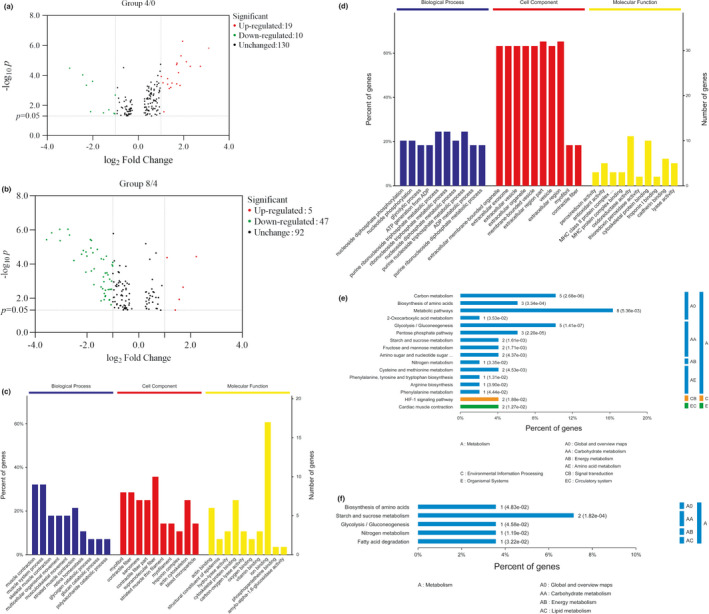
Functional analysis of differentially abundant proteins at different aging stages of Tan lamb. (a) The volcano plot showing the differentially abundant proteins in Group 4/0; (b) The volcano plot showing the differentially abundant proteins in Group 8/4; (c) The results of GO annotation of Group 4/0; (d) The results of GO annotation of Group 8/4; (e) The results of KEGG annotation of Group 4/0; (f) The results of KEGG annotation of Group 8/4

The identification and quantitation of differentially abundant proteins of Tan lamb muscles aged for days 0, 4, and 8 at 4 ºC were performed with iTRAQ‐based proteomics. The results showed that there were 29 and 52 differentially abundant proteins in Group 4/0 and Group 8/4, respectively, mainly metabolic enzymes, structural proteins, transporter proteins, stress proteins, and others (Table 1). From day 0 to day 4 postmortem, there were 19 up‐regulated and 10 down‐regulated differentially abundant proteins. From day 4 to day 8 postmortem, there were 5 up‐regulated and 47 down‐regulated differentially abundant proteins (Figure 3a,b). Among the metabolic enzymes, glycogen phosphorylase, enolase, nucleoredoxin, and aconitase 1 were up‐regulated from day 0 to day 4, and cytochrome c oxidase subunit 2 from day 4 to day 8.

**TABLE 1:** The identified differentially abundant proteins in Group 4/0 and Group 8/4 in Tan lamb

Group	ID	Protein name	FC	*P* value
4/0	O18751	Glycogen phosphorylase, muscle form	3.23	1.86E−05
	P68240	Hemoglobin subunit alpha−1/2	3.58	4.64E−04
	W5NQT7	Acyl carrier protein	2.45	3.79E−04
	W5NRC7	Troponin T3, fast skeletal type	6.67	2.41E−05
	W5NVR1	Calcium‐transporting ATPase	2.80	3.17E−04
	W5NYG7	Actin, cytoplasmic 1	0.21	4.44E−04
	W5NYJ1	Actin, alpha 1, skeletal muscle	0.12	3.26E−05
	W5P118	Troponin I2, fast skeletal type	2.58	7.28E−04
	W5P663	Enolase 3	3.27	1.50E−05
	W5P6W4	Myomesin 2	2.45	1.63E−04
	W5P9C1	Troponin C2, fast skeletal type	4.89	2.42E−05
	W5PAN7	Myosin light chain 3	2.70	6.25E−04
	W5PBN5	Myosin heavy chain 8	2.19	2.61E−02
	W5PF00	Calsequestrin	3.41	6.45E−05
	W5PK27	Myosin light chain 6	3.24	3.62E−04
	W5PNJ7	Nucleoredoxin	0.23	2.58E−02
	W5PQL7	Tropomyosin 2	3.69	4.87E−06
	W5PUC1	Carbonic anhydrase 3	2.02	1.16E−04
	W5PWE9	Serum albumin	3.87	5.22E−07
	W5PX04	Myosin binding protein C, fast type	4.34	1.21E−05
	W5PZ94	Aconitase 1	0.48	3.36E−02
	W5Q160	Keratin 10	0.25	2.45E−04
	W5Q611	Keratin 1	0.18	9.00E−05
	W5Q6U0	Fatty acid synthase	0.41	1.92E−02
	W5Q8M3	Amylo‐alpha−1, 6‐glucosidase, 4‐alpha‐glucanotransferase	2.13	3.04E−04
	W5Q9G8	Enoyl‐CoA delta isomerase 1	0.49	3.52E−02
	W5Q9Q5	Pantothenate kinase 4	0.35	3.10E−02
	W5QD16	Myosin light chain 1	10.50	3.39E−06
	W5QFC3	Four and a half LIM domains 3	0.50	2.02E−03
8/4	O78750	Cytochrome c oxidase subunit 2	2.14	4.12E−05
	P02190	Myoglobin	0.15	9.02E−07
	P09670	Superoxide dismutase [Cu‐Zn]	0.46	1.26E−02
	P14639	Serum albumin	0.29	2.19E−05
	A8DR93	Heat shock protein 90 alpha family class A member 1	0.44	3.10E−03
	A9YUY8	Adipocyte fatty acid‐binding protein 4	0.38	2.76E−03
	C8BKC5	Peroxiredoxin 2	0.44	4.26E−04
	W5NPN4	Heat shock protein family A (Hsp70) member 8	0.49	2.96E−03
	W5NR35	LIM domain binding 3	0.39	1.06E−03
	W5NRC7	Troponin T3, fast skeletal type	0.49	1.10E−04
	W5NU05	Myosin light chain kinase 2	0.48	2.43E−02
	W5NUE3	Peroxiredoxin 1	0.40	5.18E−03
	W5NX51	Apolipoprotein A1	0.33	1.37E−05
	W5NYG7	Actin, cytoplasmic 1	2.96	1.16E−02
	W5NYH2	Nucleoside diphosphate kinase	0.48	9.86E−04
	W5NYJ1	Actin, alpha 1, skeletal muscle	4.68	3.65E−05
	W5P1X9	Fructose‐bisphosphate aldolase	0.12	4.25E−06
	W5P2G4	Troponin C1, slow skeletal and cardiac type	0.37	2.19E−03
	W5P323	Glucose−6‐phosphate isomerase	0.24	1.94E−05
	W5P5W9	Triosephosphate isomerase	0.08	3.71E−06
	W5P663	Enolase 3	0.10	1.48E−06
	W5P9C1	Troponin C2, fast skeletal type	0.50	7.86E−05
	W5PDD8	Myozenin 1	0.50	1.28E−04
	W5PDG3	Glyceraldehyde−3‐phosphate dehydrogenase	0.41	7.88E−03
	Q28554	Glyceraldehyde−3‐phosphate dehydrogenase (fragment)	0.39	5.65E−04
	W5PDJ3	PDZ and LIM domain 3	0.30	1.90E−04
	W5PF65	Transferrin	0.37	4.91E−05
	W5PFT7	Fructose‐bisphosphatase 2	0.41	3.34E−04
	W5PIN6	L‐lactate dehydrogenase	0.18	6.18E−05
	W5PJB6	Phosphoglucomutase 1	0.39	5.28E−05
	W5PK66	Parkinsonism‐associated deglycase	0.46	7.63E−04
	W5PNJ7	Nucleoredoxin	2.67	4.89E−02
	W5PQL7	Tropomyosin 2	0.22	2.63E−05
	W5PS88	Aspartate aminotransferase	0.24	2.94E−04
	W5PUC1	Carbonic anhydrase 3	0.28	1.14E−05
	W5PVY5	Phosphoglycerate mutase	0.23	1.59E−05
	W5PWE9	Serum albumin	0.28	4.16E−06
	W5PZS7	Serpin family A member 1	0.43	3.22E−04
	W5Q0I1	Myosin binding protein C, slow type	0.42	3.44E−05
	W5Q2E1	Lumican	0.41	7.06E−03
	W5Q5Y7	Synaptopodin 2	0.41	3.36E−03
	W5Q611	Keratin 1	3.28	2.23E−03
	W5Q7C7	Proteasome subunit alpha type	0.40	1.22E−02
	W5Q7N3	PDZ and LIM domain 5	0.36	1.41E−02
	W5Q8N4	Myosin light chain 2	0.25	3.39E−05
	W5Q983	Glycerol−3‐phosphate dehydrogenase [NAD(+)]	0.27	3.66E−06
	W5QBV3	Phosphoglycerate kinase	0.28	2.53E−04
	W5QC41	Pyruvate kinase	0.12	9.25E−07
	W5QFN9	UDP‐glucose pyrophosphorylase 2	0.46	3.01E−03
	W5QFQ1	Malate dehydrogenase	0.16	1.99E−06
	W5QG04	Calsequestrin	0.46	1.21E−02
	W5QH56	Alpha−2‐HS‐glycoprotein	0.30	1.61E−03

The functional analysis of differentially abundant proteins was preformed through GO and KEGG annotation. The results of GO annotation showed that from day 0 to day 4, the identified proteins in Tan lamb responded to muscle contraction, muscle system process, and multicellular organismal movement in terms of biological process, served as structural proteins in terms of cell component, and functioned in binding, catalytic activity, and structural molecular activity in terms of molecular function. From day 4 to day 8, the identified proteins in Tan lamb responded for muscle contraction, muscle movement, and catabolic process in terms of biological process, served as structural proteins and microparticle in terms of cell component, and functioned in binding, catalytic activity, and structural molecular activity in terms of molecular function (Figure 3c,d). The results of KEGG pathway annotation showed that the differentially abundant proteins were involved in metabolism from day 0 to day 4, and in metabolism, environmental information processing, and organismal systems, including carbohydrate metabolism, amino acids metabolism, and signaling pathway from day 4 to day 8 (Figure 3e,f).

The differentially abundant proteins relating to Tan lamb flavor precursors are listed in Table 2. These proteins are mainly structural proteins, metabolic enzymes, and their co‐factors, such as troponin, actin, tropomyosin, myosin light chain, myosin heavy chain, glyceraldehyde‐3‐phosphate dehydrogenase, phosphoglycerate mutase, pyruvate kinase, and UDP‐glucose pyrophosphorylase 2. Significantly, glycogen phosphorylase, amylo‐alpha‐1, 6‐glucosidase, and 4‐alpha‐glucanotransferase were all involved in glycogen degradation (*p* < .05). Calcium‐transporting ATPase, calsequestrin, and carbonic anhydrase 3 were found to increase before day 4, which involved regulation of ion homeostasis (*p* < .05).

**TABLE 2:** The differentially abundant proteins closely relating to water‐soluble flavor precursors in Tan lamb during postmortem aging

Protein ID	Protein name	4/0 Fold Change	8/4 Fold Change
O18751	Glycogen phosphorylase, muscle form	3.23	‐
W5NQT7	Acyl carrier protein	2.45	0.71
W5NRC7	Troponin T3, fast skeletal type	6.67	0.49
W5NYG7	Actin, cytoplasmic 1	0.21	2.96
W5NYJ1	Actin, alpha 1, skeletal muscle	0.12	4.68
W5P118	Troponin I2, fast skeletal type	2.58	0.52
W5P663	Enolase 3	3.27	0.10
W5P6W4	Myomesin 2	2.45	‐
W5P9C1	Troponin C2, fast skeletal type	4.89	0.50
W5PAN7	Myosin light chain 3	2.7	‐
W5PBN5	Myosin heavy chain 8	2.19	‐
W5PF00	Calsequestrin	3.41	0.61
W5PK27	Myosin light chain 6	3.24	0.66
W5PQL7	Tropomyosin 2	3.69	0.22
W5PX04	Myosin binding protein C, fast type	4.34	1.36
W5PZ94	Aconitase 1	0.48	‐
W5Q6U0	Fatty acid synthase	0.41	‐
W5Q8M3	Amylo‐alpha−1, 6‐glucosidase, 4‐alpha‐glucanotransferase	2.13	‐
W5Q9G8	Enoyl‐CoA delta isomerase 1	0.49	‐
W5QD16	Myosin light chain 1	10.5	0.52
A9YUY8	Adipocyte fatty acid‐binding protein 4	1.61	0.38
W5NU05	Myosin light chain kinase 2	‐	0.48
W5NYH2	Nucleoside diphosphate kinase	1.39	0.48
W5P1X9	Fructose‐bisphosphate aldolase	1.33	0.12
W5P2G4	Troponin C1, slow skeletal and cardiac type	1.67	0.37
W5P323	Glucose−6‐phosphate isomerase	1.39	0.24
W5P5W9	Triosephosphate isomerase	1.95	0.08
W5PDD8	Myozenin 1	1.48	0.50
Q28554	Glyceraldehyde−3‐phosphate dehydrogenase (fragment)	‐	0.39
W5PDG3	Glyceraldehyde−3‐phosphate dehydrogenase	‐	0.41
W5PFT7	Fructose‐bisphosphatase 2	1.29	0.41
W5PIN6	L‐lactate dehydrogenase	1.46	0.18
W5PJB6	Phosphoglucomutase 1	1.48	0.39
W5PS88	Aspartate aminotransferase	‐	0.24
W5PVY5	Phosphoglycerate mutase	1.37	0.23
W5Q0I1	Myosin binding protein C, slow type	1.21	0.42
W5Q8N4	Myosin light chain 2	1.76	0.25
W5Q983	Glycerol−3‐phosphate dehydrogenase [NAD (+)]	1.45	0.27
W5QBV3	Phosphoglycerate kinase	‐	0.28
W5QC41	Pyruvate kinase	1.49	0.12
W5QFN9	UDP‐glucose pyrophosphorylase 2	‐	0.46
W5QFQ1	Malate dehydrogenase	1.92	0.16
W5PDG3	Glyceraldehyde−3‐phosphate dehydrogenase	‐	0.41

Note

“‐” means no significant difference in the corresponding group.

**FIGURE 4:**
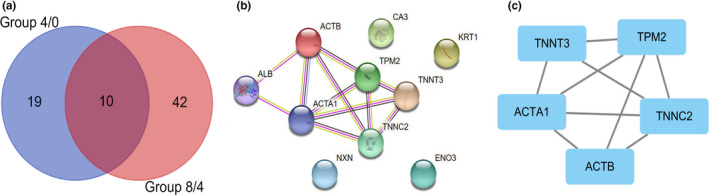
PPI network of common differentially abundant proteins in Tan lamb during postmortem aging (a) Common differentially abundant proteins in pair comparison; (b) Protein–protein interaction (PPI) network of common differentially abundant proteins constructed via STRING (http://www.string‐db.org/); (c) Core differentially abundant proteins identified via MCODE in Cytoscape

For the overlapping differentially abundant proteins in different comparison groups as indicated by Venn diagram, 10 were identified as common proteins between two comparison groups (Figure 4a). The interaction network of 10 differentially abundant proteins in both groups 4/0 and 8/4 was constructed using String and analyzed by the MCODE plug‐in in Cytoscape (Figure 4b,c). It was found that actin (cytoplasmic 1), tropomyosin 2, Troponin C2 (fast skeletal type), actin (alpha 1), and troponin T3 (fast skeletal type), all as structural proteins, played important regulative roles in the formation of flavor precursor of Tan lamb meat during postmortem aging.

### 3.3 Identification of differential metabolites of Tan lamb at different aging stages

The identification and quantitation of differential metabolites of muscles aged for 0, 4, and 8 days at 4 ºC were performed by GC×GC‐TOF‐MS. The results showed that there were 22 and 21 target differential metabolites in Group 4/0 and Group 8/4, respectively. The differential metabolites were mainly the degradation products of nucleic acid, carbohydrates, free amino acids, and their derivatives (Table 3).

**TABLE 3:** The identified differential metabolites in Group 4/0 and Group 8/4 in Tan lamb

Group	Metabolites	Quant Mass	RT (min)	VIP	*p* value	FC
4/0	Inosine	230	26.03	2.14	2.41E−05	1.36
	D‐Glyceric acid	189	10.62	2.26	8.29E−05	2.36
	Glucose−6‐phosphate	387	23.97	2.03	2.98E−04	1.96
	Tetracosane	57	22.69	1.18	8.61E−04	3.19
	Lactose	217	17.96	1.85	1.89E−03	1.56
	Succinic acid	247	10.49	1.80	2.19E−03	1.49
	Fructose−6‐phosphate	459	24.03	1.86	2.36E−03	1.70
	3‐Hydroxy‐L‐proline	86	7.17	1.77	3.36E−03	2.78
	*N*‐epsilon‐Acetyl‐L‐lysine	200	10.13	1.75	4.01E−03	0.75
	Ribulose−5‐phosphate	387	24.29	1.76	4.56E−03	1.66
	Aspartic acid	232	13.21	2.02	4.63E−03	3.25
	Hypoxanthine	265	18.05	1.07	5.62E−03	1.93
	Threitol	58	25.29	1.60	1.25E−02	0.76
	3‐Hydroxybutyric acid	117	8.64	1.45	1.68E−02	0.70
	Cis‐gondoic acid	57	22.60	1.33	2.09E−02	0.20
	Glycerol	205	9.91	1.36	3.67E−02	1.26
	Pyruvic acid	174	7.46	1.56	3.89E−02	0.64
	Ribose−5‐phosphate	315	23.64	1.10	4.27E−02	1.82
	Methionine	176	13.32	1.30	4.37E−02	1.49
	Tyrosine	226	17.21	1.33	4.48E−02	0.73
	Citric acid	273	17.79	1.15	4.72E−02	1.51
	Nicotinoylglycine	207	7.28	1.26	4.81E−02	1.34
8/4	Inosine	230	26.03	2.14	2.41E−05	1.51
	Tyrosine	226	17.21	2.03	3.55E−04	1.82
	Methylmalonic acid	218	8.37	1.86	1.28E−03	0.86
	Gluconic acid	73	20.82	1.85	1.30E−03	1.60
	Lactic acid	117	7.39	1.87	1.49E−03	0.94
	Methionine	176	13.32	1.95	3.89E−03	2.66
	Threitol	58	25.29	1.70	7.26E−03	0.71
	Maleamate	151	7.89	1.56	9.30E−03	0.87
	*N*‐epsilon‐Acetyl‐L‐lysine	200	10.13	1.56	1.20E−02	0.78
	O‐Phosphorylethanolamine	73	17.30	1.59	1.32E−02	1.28
	Aspartic acid	232	13.21	1.62	2.14E−02	1.90
	Phenylalanine	218	14.97	1.53	2.15E−02	2.03
	Valine	144	9.31	1.42	2.46E−02	1.52
	Hypoxanthine	265	18.05	1.44	2.50E−02	1.14
	*N*‐alpha‐Acetyl‐L‐ornithine	154	12.49	1.42	2.56E−02	0.89
	Aminomalonic acid	218	12.54	1.40	3.05E−02	1.34
	Isoleucine	158	10.25	1.41	3.14E−02	1.56
	Threonine	219	11.36	1.38	3.30E−02	1.33
	Serine	204	11.02	1.35	3.49E−02	1.47
	D‐Glyceric acid	189	10.62	1.28	3.93E−02	1.42
	Sucrose	361	26.47	1.20	4.87E−02	0.47

The results from KEGG pathway annotation of target differential metabolites showed that the differential metabolites were mainly involved in carbon metabolism, citrate cycle (TCA cycle), glycolysis/gluconeogenesis, pentose phosphate pathway, aminoacyl‐tRNA biosynthesis, and amino acids metabolism from day 0 to day 4 and in purine metabolism, amino acids metabolism, biosynthesis, and degradation from day 4 to day 8 (Table 4).

**TABLE 4:** The results of KEGG pathway annotation of identified differential metabolites in Group 4/0 and Group 8/4 of Tan lamb

Group	KEGG pathway (*Ovis aries*)	Matched objects
4/0	Carbon metabolism	Pyruvate, Succinate, L‐Aspartate, D‐Fructose 6‐phosphate, D‐Ribose 5‐phosphate, Citrate, D‐Glycerate, alpha‐D‐Glucose 6‐phosphate
	2‐Oxocarboxylic acid metabolism	Pyruvate, L‐Aspartate, L‐Methionine, L‐Tyrosine, Citrate
	Biosynthesis of amino acids	Pyruvate, L‐Aspartate, L‐Methionine, L‐Tyrosine, D‐Ribose 5‐phosphate, Citrate
	Glycolysis/Gluconeogenesis	Pyruvate, alpha‐D‐Glucose 6‐phosphate
	Citrate cycle (TCA cycle)	Pyruvate, Succinate, Citrate
	Pentose phosphate pathway	Pyruvate, D‐Ribose 5‐phosphate, D‐Glycerate, alpha‐D‐Glucose 6‐phosphate
	Pentose and glucuronate interconversions	Pyruvate, Glycerol
	Galactose metabolism	D‐Fructose 6‐phosphate, Glycerol, Alpha‐D‐Glucose 6‐phosphate
	Pyruvate metabolism	Pyruvate, Succinate
	Glyoxylate and dicarboxylate metabolism	Pyruvate, Succinate, Citrate, D‐Glycerate
	Glycerolipid metabolism	Glycerol, D‐Glycerate
	Alanine, aspartate, and glutamate metabolism	Pyruvate, Succinate, L‐Aspartate, Citrate
	Glycine, serine, and threonine metabolism	Pyruvate, L‐Aspartate, D‐Glycerate
	Cysteine and methionine metabolism	Pyruvate, L‐Aspartate, L‐Methionine
	Lysine degradation	Succinate, N6‐Acetyl‐L‐lysine
	Tyrosine metabolism	Pyruvate, Succinate, L‐Tyrosine
	Phenylalanine metabolism	Pyruvate, Succinate, L‐Tyrosine
	Nicotinate and nicotinamide metabolism	Pyruvate, Succinate, L‐Aspartate, Nicotinate
	Aminoacyl‐tRNA biosynthesis	L‐Aspartate, L‐Methionine, L‐Tyrosine
8/4	Aminoacyl‐tRNA biosynthesis	L‐Aspartate, L‐Methionine, L‐Phenylalanine, L‐Tyrosine, L‐Valine, L‐Threonine, L‐Isoleucine
	2‐Oxocarboxylic acid metabolism	L‐Aspartate, L‐Methionine, L‐Phenylalanine, L‐Tyrosine, L‐Valine, L‐Isoleucine
	Valine, leucine, and isoleucine biosynthesis	L‐Valine, L‐Threonine, L‐Isoleucine
	Glycine, serine, and threonine metabolism	L‐Aspartate, L‐Threonine, D‐Glycerate
	Valine, leucine, and isoleucine degradation	L‐Valine, L‐Isoleucine, Methylmalonate
	Purine metabolism	IMP, Hypoxanthine
	Cysteine and methionine metabolism	L‐Aspartate, L‐Methionine
	Pantothenate and CoA biosynthesis	L‐Aspartate, L‐Valine
	Phenylalanine, tyrosine, and tryptophan biosynthesis	L‐Phenylalanine, L‐Tyrosine
	Phenylalanine metabolism	L‐Phenylalanine, L‐Tyrosine
	Pentose phosphate pathway	D‐Gluconate, D‐Glycerate

### 3.4 Joint analysis of proteomics and metabolomics

**FIGURE 5:**
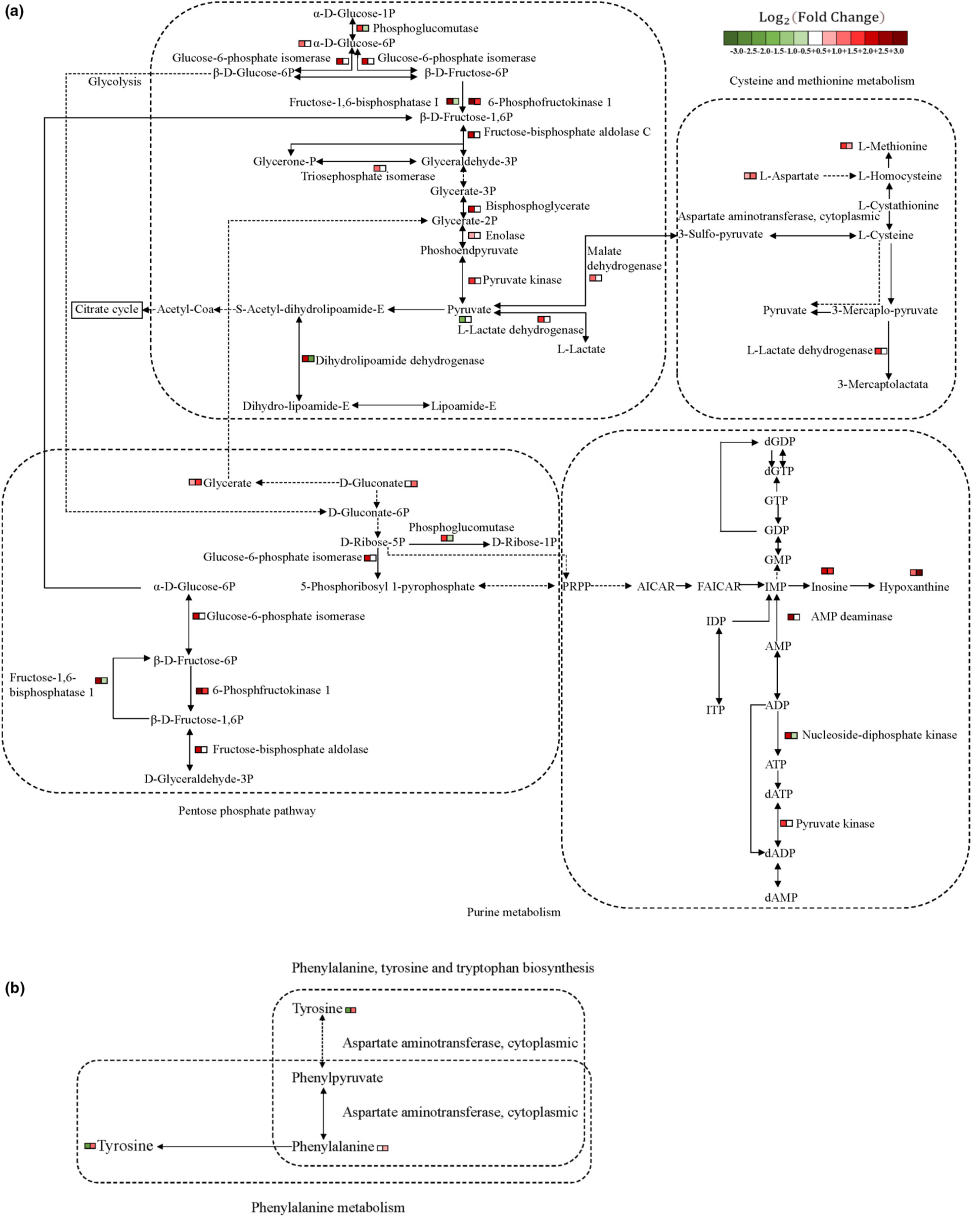
Pathway map visualizing proteomic and metabolomic changes in Tan lamb during the period of aging. (a) Integrated proteomic and metabolomic changes in glycolysis/gluconeogenesis, pentose phosphate pathway, purine metabolism, and cysteine and methionine metabolism in Tan lamb; (b) Pathway map visualizing proteomic and metabolomic changes in phenylalanine metabolism and phenylalanine, tyrosine, and tryptophan biosynthesis. Note: The color of □ represents the change of the corresponding metabolite or protein, red for up‐regulation, green for down‐regulation, and the depth of the color for the degree of change

To elucidate the effects of postmortem aging on water‐soluble flavor precursors of Tan lamb meat, the KEGG pathways were selected as the carrier to map the differentially abundant proteins and differential metabolites. The results showed that glycolysis/gluconeogenesis was the main metabolic pathways involving the target metabolites and proteins mainly at the early aging stage (day 0 to 4). Meanwhile, cysteine and methionine metabolism, phenylalanine, tyrosine and tryptophan metabolism, phenylalanine metabolism, pentose phosphate pathway, and purine metabolism were the main metabolic pathways involving the target metabolites and proteins mainly at the later aging stage (day 4 to 8). The mainly enriched pathways were present succinctly (Figure 5) to show the relationship between differential metabolites and differentially abundant proteins intuitively. The activity of metabolic enzymes regulated the accumulation of carbohydrate‐related metabolites.

**FIGURE 6:**
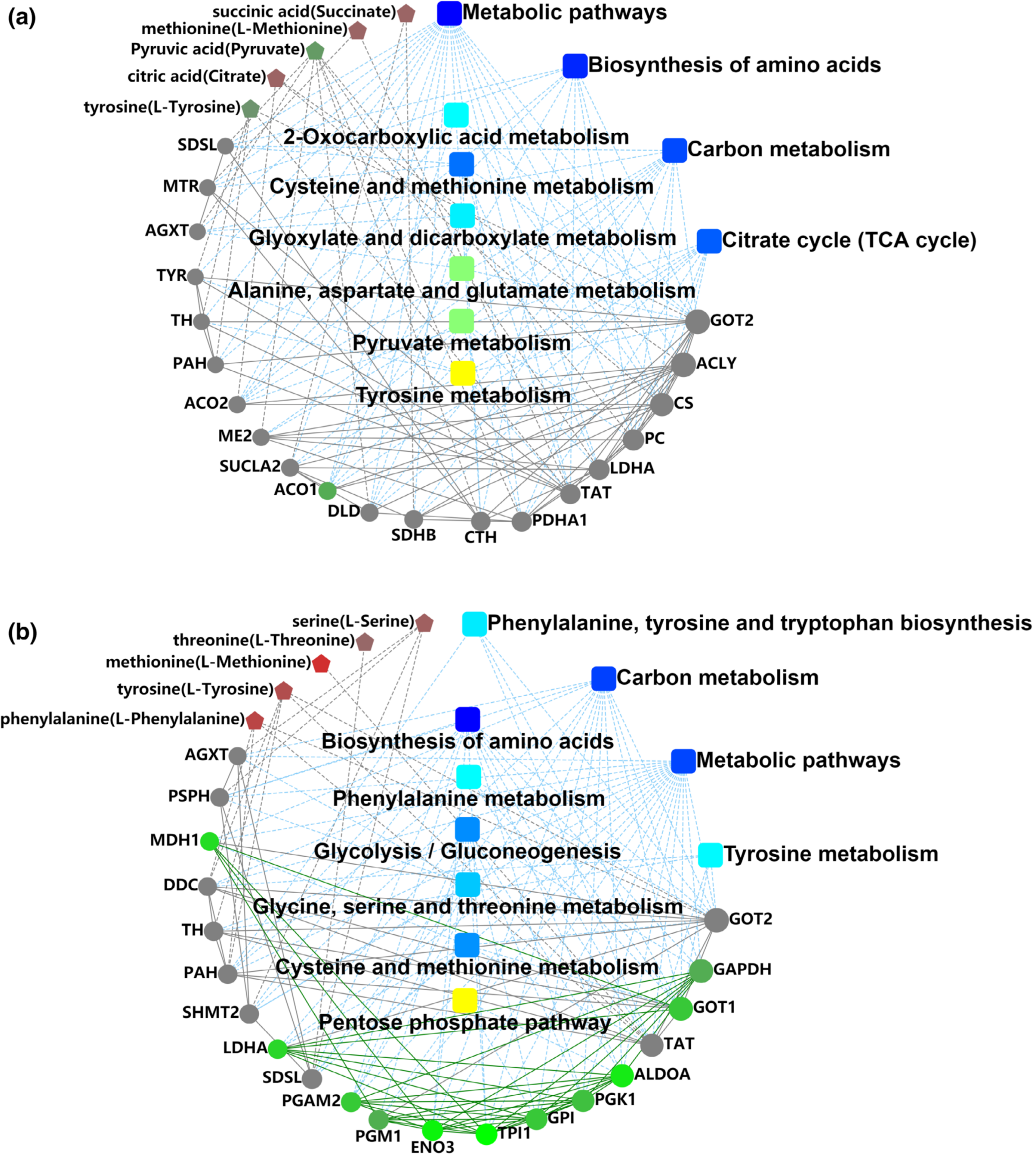
The regulative relationships between differentially abundant proteins and differential metabolites in Tan lamb in pair comparison during postmortem aging (a) The results of Group 4/0; (b) The results of Group 8/4. Note: The icon □ is for GO/KEGG terms, ○ for proteins/genes, and ⌂ for metabolites, and the color green is for down‐regulation, and red for up‐regulation. The green line between icons indicates the inhibition between proteins and metabolites, red line indicates the activation between proteins and metabolites, and dotted lines indicate GO pathway and KEGG pathway

The interaction networks were built (Figure 6) to further illustrate the regulative relationships between differentially abundant proteins and differential metabolites. A complex regulative relationship was observed between the identified differentially abundant proteins and differential metabolites.

## 4 DISCUSSION

### 4.1 Activation of postmortem glycolysis contributing to muscle pH

From day 0 to day 4 postmortem, glycolysis/gluconeogenesis was the main metabolic pathways involving the target metabolites and proteins. The activity of LDH was observed significantly increasing from day 0 to day 4 postmortem, and decreasing from day 4 to day 8 (*p* < .05), suggesting that under postmortem anaerobic conditions, energy metabolism turned to anoxic degradation, LDH was activated, and subsequently glycogen was converted into lactic acid. This was one of the reasons why muscle pH decreased before day 4. Numerous studies have revealed that hypoxic ischemia leads to increased activity of glycolytic enzymes in muscle cells at the early postmortem stages, and subsequently promotes the consumption of glycogen, decreases muscle pH, and accelerates the aging of muscle (Apaoblaza et al., 2015; England et al., 2015; Hardie, 2011; Lametsch et al., 2002). The result of the present study is consistent with the previous studies.

Pyruvate, one of the important metabolites of glycolysis, decreased before day 4 postmortem, probably due to rapid/excessive glycolysis converting pyruvate to lactic acid (Table 3). Pyruvate kinase increased from day 0 to day 4 (FC =1.49, Table 2), while higher pyruvate kinase concentrations and activities are hallmarks of a greater glycolytic metabolism in the muscle tissue (Baldassini et al., 2015). All of these data indicate that glycolysis was activated in Tan lamb muscles within 4 days postmortem. The concentration of enolase 3 was found to increase before day 4 (Table 1). The up‐regulation of enolase 3 during postmortem storage was consistent with Holstein cattle *semitendinosus* muscle in which there was an increase of this enzyme after 4 days of aging (Yu et al., 2017). Furthermore, triosephosphate isomerase was found to increase before day 4 and decrease from day 4 to day 8 (Table 2). During postmortem storage of *semitendinosus* muscles of Chinese Luxi yellow cattle, triosephosphate isomerase showed 79.34‐fold increase in day 10 versus. day 0 set (Wu et al., 2015). The report of Zuo et al. highlighted that triosephosphate isomerase may have the potential to serve as biological markers for water‐holding capacity prediction of yak meat (Zuo et al., 2016). Therefore, glycolysis enzymes contribute to the accumulation of flavor‐related carbohydrates, and simultaneously, affect the development of other quality traits of meat.

Within 4 days of aging, the content of fructose‐6‐phosphate and glucose‐6‐phosphate increased, which could be due to the activation of glycolysis (Table 3). The research of Meinert et al. showed that glucose‐6‐phosphate generated a large number of volatiles in pork upon heating (Meinert et al., 2009). These water‐soluble metabolites contribute to cooked meat flavor, to a greater or lesser extent, as they do or as precursors do in the Maillard reactions.

Muscle pH is one of the most important indexes which influence meat quality by affecting protein characteristics and endogenous enzyme activity. In this study, the pH value considerably decreased in the first 4 days, and increased from day 4 to day 8 (*p* <.05). Previous studies have shown that the pH value of livestock muscles can drop to 5.4 ~ 6.3 at the early stage of postmortem aging (Sánchez‐Macías et al., 2019; Ylä‐Ajos et al., 2006), which is consistent with the result of the present study. It has been explained in a large number of studies that the pH declines in the early postmortem muscle cells due to the increase in the H^+^ content from postmortem ATP hydrolysis and the accumulation of lactate in muscles (Shen et al., 2014).

From day 4 to day 8 postmortem, fructose‐bisphosphate aldolase, glucose‐6‐phosphate isomerase, triosephosphate isomerase, enolase 3, glyceraldehyde‐3‐phosphate dehydrogenase, fructose‐bisphosphatase 2, L‐lactate dehydrogenase, phosphoglucomutase 1, phosphoglycerate mutase, phosphoglycerate kinase, and pyruvate kinase decreased (Tables 1 and 2), which could be attributable to the gradual degradation of the proteins in Tan lamb muscles with the extension of postmortem time. The decrease of fructose‐bisphosphate aldolase, phosphoglycerate mutase, phosphoglucomutase 1, phosphoglycerate kinase, and enolase 3 during postmortem storage was consistent with that in Hengshan goat *longissimus lumborum* muscles stored at −18°C for 30 days (Jia, Zhang, et al., 2021).

The large number of nonvolatile compounds produced by glycolysis metabolites contribute to meat flavor upon heating, under the domination of the key enzymes of glycolysis, which is consistent with the result of Toldrá and Flores (2000). Glycolysis in postmortem Tan lamb muscles directly affecting variations in metabolic enzymes triggers the accumulation of flavor‐related carbohydrates.

### 4.2 Degradation of structural proteins leading to accumulation of amino acids

The compositions and contents of amino acids in meat are one of the criteria for assessment of nutritional value, and one of the most important factors influencing meat flavor. In the process of postmortem aging, the structural proteins actin, actin α 1, troponin C1, C2, troponin T3, troponin I2, myozenin1, and myosin light chain 1, 2, and 6 in Tan lamb changed significantly and released plenty of amino acids through hydrolysis with the extension of postmortem time. The content of proteasome subunit alpha type (FC =0.40, *p* < .05), proteasome 26S subunit, ATPase 1 (FC =0.83, *p* < .05), proteasome 26S subunit, and non‐ATPase 3 (FC =0.52, *p* < .05) decreased from day 4 to day 8 postmortem.

Calcium‐transporting ATPase, calsequestrin, and carbonic anhydrase 3 were found to increase before day 4, which involved regulation of ion homeostasis. The Ca^2+^‐ATPase mediated the transport of Ca^2+^ across the plasma membrane of muscle cells. Calsequestrin binds approximately 50 mol of Ca^2+^ per mol of protein. The up‐regulation of these proteins indicates that before day 4 postmortem, a large number of ion exchange could be occurring in Tan lamb muscle cells. Sierra et al. summarized that a large number of ion exchange in the early postmortem muscle cells leads to calcium homeostasis imbalance (Sierra & Oliván, 2013), and subsequently the activation of calpain‐1 and caspase‐3 to hydrolyze the myofibrillar proteins (Ahmed et al., 2015; Huang et al., 2009). Therefore, the content of metabolites such as 3‐hydroxy‐L‐proline, aspartic acid, and methionine increased from day 0 to day 4, and aspartic acid, serine, threonine, tyrosine, phenylalanine, and D‐phenylalanine from day 4 to day 8. Most of the free amino acids accumulated at day 8 indicated the progression of proteolysis of Tan lamb during aging. The changes in metabolite profiling during pork postmortem aging showed that phenylalanine, serine, and threonine accumulated after 14 days postmortem (Tamura et al., 2022). The variation tendency of these free amino acids in the present study is consistent with that in the previous studies. Besides, proteomics was used to investigate the proteolysis of myofibrillar proteins occurring in different horse muscles. It was observed that compared to *longissimus lumborum* and *semimembranosus* muscles, more extensive proteolysis occurred in *semitendinosus* muscle during postmortem aging (della Malva et al., 2019).

Amino acids have many cellular physiological functions. Aspartic acid, one of the components of cell wall peptidoglycan, regulates intracellular pH through the decarboxylase pathway. L‐serine competes with glycolysis to reduce energy and substrate formation. The complex physiological functions of amino acids may play important roles in regulating the aging process. As important flavor substances in meat, free amino acids react with sugars and/or other substances to form the flavor of meat upon heating (Lee et al., 2016; Mottram, 1998). Research highlights that cystine, cysteine, and methionine can be decomposed to produce hydrogen sulfide, methyl mercaptan, and thioformaldehyde, the important precursors of volatile aroma compounds (Mottram, 1998). Therefore, the accumulation of amino acids during postmortem aging plays crucial roles in improving meat flavor.

### 4.3 Degradation of ATP contributing to nucleotides accumulation during postmortem aging

Nucleotides mainly come from ATP metabolism in Tan lamb. ATP is the energy substance of metabolic reactions in both living animals and early postmortem muscles. The activation of glycolysis at the early stage of aging is to maintain ATP levels in postmortem Tan lamb muscles. However, due to the limited energy production capacity of glycolysis and intensified catabolism, the residual ATP was degraded. In the process of Tan lamb aging, nucleotides were degraded into adenosine diphosphate, adenosine, inosine, and hypoxanthine under the action of a series of enzymes. Nucleoside‐diphosphate kinase was observed significantly increasing from day 0 to day 4, and decreasing from day 4 to day 8 (Table 2). Both inosine and hypoxanthine increased significantly during the whole period of aging (Table 3), which could be due the degradation of ATP into IMP and AMP, and further into inosine. The results of this study were consistent with previous research (Aliani et al., 2013). The GC–TOF–MS‐based metabolomic was used in a recent study to investigate the taste‐related metabolites in aged pork. The hypoxanthine was found increasing after 14‐day aging (Tamura et al., 2022), which is consistent with the results of the present study. IMP, an important flavor substance giving umami taste in meat, has degradation products as important constituents in formation and development of meat flavor (Madruga et al., 2010). The bitterness in meat comes from hypoxanthine. Previously, it was found that the concentration of IMP dropped with a simultaneous increase in the concentrations of inosine, hypoxanthine, and ribose in beef with aging time (Tikk et al., 2006). The increase of inosine and hypoxanthine at day 4 and day 8 of aging in the present study is consistent with that in the previous study. Therefore, the mechanism of energy metabolism and ATP degradation in postmortem muscles is of great importance to meat flavor.

## 5 CONCLUSIONS

In conclusion, the results of this study highlight the protein and metabolic changes occurring in Tan lamb muscles during postmortem aging. Muscle pH considerably decreased from day 0 to day 4, and increased from day 4 to day 8 (*p* < .05). The activity of LDH significantly increased from day 0 to day 4, and decreased from day 4 to day 8 (*p* < .05). Postmortem glycolysis was activated in 4 days, which directly affected variations in metabolic enzymes and triggered the accumulation of flavor‐related carbohydrates. The free amino acids accumulated due to hydrolysis of structural proteins, with 3‐hydroxy‐L‐proline, aspartic acid, and methionine increasing from day 0 to day 4, and aspartic acid, serine, threonine, tyrosine, phenylalanine, and D‐phenylalanine from day 4 to day 8. The inosine and hypoxanthine accumulated due to the degradation of ATP. The abundance of these water‐soluble flavor precursors led to the change of meat flavor upon heating. Particularly, the structural proteins, actin (cytoplasmic 1), tropomyosin 2, Troponin C2 (fast skeletal type), actin alpha 1, and troponin T3 (fast skeletal type) could serve as the candidate predictors for monitoring of meat flavor precursor during postmortem storage.

## CONFLICT OF INTERESTS

All authors have declared no conflict of interests.

## AUTHOR CONTRIBUTION

**Chen Ji:** Data curation (lead); Methodology (lead); Writing – original draft (lead). **Liqin You:** Conceptualization (lead); Methodology (supporting); Writing – review & editing (lead). **ruiming luo:** Funding acquisition (lead); Supervision (lead).

## Data Availability

Data available on request from the authors.
